# Targeted Drug Delivery to the Central Nervous System Using Extracellular Vesicles

**DOI:** 10.3390/ph15030358

**Published:** 2022-03-15

**Authors:** Lina Zhou, Sunitha Kodidela, Sandip Godse, Stacey Thomas-Gooch, Asit Kumar, Babatunde Raji, Kaining Zhi, Harry Kochat, Santosh Kumar

**Affiliations:** 1 Department of Pharmaceutical Sciences, University of Tennessee Health Science Center, 881 Madison Ave, Memphis, TN 38163, USA; lzhou13@uthsc.edu (L.Z.); sgodse@uthsc.edu (S.G.); sthoma36@uthsc.edu (S.T.-G.); akumar23@uthsc.edu (A.K.); 2 Plough Center for Sterile Drug Delivery Solutions, University of Tennessee Health Science Center, 208 South Dudley Street, Memphis, TN 38163, USA; braji@uthsc.edu (B.R.); kzhi@uthsc.edu (K.Z.); hkochat@uthsc.edu (H.K.)

**Keywords:** BBB, CNS, target drug delivery, extracellular vesicles, nanoparticles, drug-loading, brain

## Abstract

The blood brain barrier (BBB) maintains the homeostasis of the central nervous system (CNS) and protects the brain from toxic substances present in the circulating blood. However, the impermeability of the BBB to drugs is a hurdle for CNS drug development, which hinders the distribution of the most therapeutic molecules into the brain. Therefore, scientists have been striving to develop safe and effective technologies to advance drug penetration into the CNS with higher targeting properties and lower off-targeting side effects. This review will discuss the limitation of artificial nanomedicine in CNS drug delivery and the use of natural extracellular vesicles (EVs), as therapeutic vehicles to achieve targeted delivery to the CNS. Information on clinical trials regarding CNS targeted drug delivery using EVs is very limited. Thus, this review will also briefly highlight the recent clinical studies on targeted drug delivery in the peripheral nervous system to shed light on potential strategies for CNS drug delivery. Different technologies engaged in pre- and post-isolation have been implemented to further utilize and optimize the natural property of EVs. EVs from various sources have also been applied in the engineering of EVs for CNS targeted drug delivery in vitro and in vivo. Here, the future feasibility of those studies in clinic will be discussed.

## 1. Introduction

The majority of Food and Drug Administration (FDA) and World Health Organization (WHO)-approved drugs effectively treat the peripheral pathology or symptoms of a disease. However, they are ineffective or achieve suboptimal responses while treating the pathology or symptoms related to the brain [1,2]. The suboptimal therapeutic concentration of drugs in the brain is mainly due to the relative impermeability of the blood-brain barrier (BBB) to most pharmacological agents [1,2]. The lack of BBB permeability is attributed to both a tight BBB junction and efflux of these drugs, primarily via P-glycoprotein (P-gp) [3,4,5].

Nanotechnology has emerged to overcome unmet drug delivery barriers and to achieve site targeted drug delivery. It has contributed substantially to developing nanocarriers to treat various diseases in recent decades [6,7]. In particular, liposomes have attracted much attention in drug encapsulation and delivery, and many lab scale liposome drug preparations have been transformed into clinical formulations [8,9]. These synthetic vesicles are attached to different ligands to aid in the efficient cellular binding, uptake, and intracellular processing required for targeted delivery of the cargo [10,11]. Nevertheless, these ligands on drug carriers can trigger an immune response [12,13,14,15]. Further, the difficulty in the clearance or biodegradation of these nanoparticles (NPs), which could lead to neurotoxicity, limits their use as drug delivery vehicles [16,17,18].

Recently, extracellular vesicles (EVs) started emerging as a natural carrier system for the delivery of therapeutics [19,20]. EVs, nano-sized membranous vesicles, are released by many types of cells. The natural biocompatibility, stability, permeability across several natural barriers, as well as their inherent homing aptitudes facilitates EVs’ potential for targeted therapeutic drug delivery [21,22]. They have an extraordinary ability to deliver therapeutics to target cells due to the expression of various adhesive proteins, LFA1/ICAM1, on their surface [23]. It has been reported that EVs are able to cargo small molecules, e.g., nucleic acids across the BBB to mitigate the symptoms of several central nervous system (CNS) diseases, such as Parkinson’s disease(PD) and brain cancer [24,25]. In addition, EV-based therapeutics show promising results in various stages of clinical trials, which we will discuss later in this review. EVs have been engineered using multiple methods such as surface, functional and chemical modifications to maximize their targeted effect and minimize the off-target effects [26,27,28,29].

**Figure 1:**
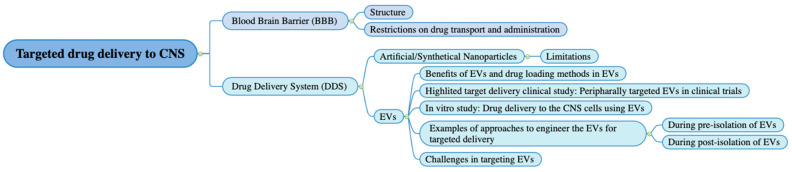
A schematic coverage of this review. We first discuss a brief explanation of the structure of the blood brain barrier (BBB) and its effect on drug permeability followed by the limitations of the current nanoformulation in drug delivery. Afterwards, we introduce EVs for targeted drug delivery, in which we list several clinical studies on targeted delivery in the peripheral system. Furthermore, we focus on engineering extracellular vesicles (EVs) for targeted delivery in the central nervous system (CNS) with four examples that are discussed in detail. Finally, we provide some limitations regarding an EV-based drug delivery system (DDS) during manufacturing.

In a recent review, Yelamanchili et al. described EVs’ potential role as drug delivery vehicles to the CNS [30]. This review will expand the discussion on the benefits of EVs in drug delivery systems over other nanoparticle-based systems. We intend to describe the strategies used to achieve targeted delivery systems with EVs and the current challenges in achieving targeted delivery using EVs and their applications in treating diseases. We will expand our thoughts as shown in Figure 1.

## 2. BBB Structure, Drug Transportation, and Administration

The structure of the BBB is mainly composed of capillary endothelial cells (ECs), a basement membrane, neuroglial membrane, astrocytes, and glial podocytes [31]. The BBB is an intricate vasculature network in the CNS with a circumscribed rate of transcytosis as well as conditional paracellular permeability. Only under certain pathological conditions will the integrity of the BBB structure be disrupted, which allows drug delivery into the CNS [32].

Despite neurological diseases having a high incidence, these diseases have some of the highest therapeutic failure rates because the BBB has not only been a physical barrier but also a transport interface for drug delivery [33]. The homeostasis of the CNS is critically dependent on the function and structural integrity of the BBB. The brain and spinal cord are permeated by cerebral capillaries and lined with microvascular ECs [34]. The ECs that are located in the CNS are uniquely distinctive from other EC locations given that CNS ECs have a flattened shape, have fewer caveolae at the luminal surface, have an increased quantity of mitochondria, and express interendothelial tight junctions [35]. These are some of the characteristics that are essential for the BBB to control transportation.

This biological barrier utilizes different highly selective cells and structures to allow nutrients or to prevent toxins entry into the brain via the influx and efflux of endogenous and exogenous molecules [35,36]. In the case of ECs and tight junctions, the tight junctions form a wall-like structure around the ECs thus preventing molecules from passing between ECs. On the contrary, ECs do allow simple diffusion of oxygen and carbon dioxide. A variety of therapeutic molecules can traverse via simple diffusion regulated by lipids, however, these molecules must meet parameters; for instance, unionized and molecular weight < 400 Da [34]. Many small molecules, including pharmaceuticals, are not compatible to cross the BBB alone. For such circumstances, extracellular vesicles (EVs) are currently being investigated as biologically compatible couriers into the brain. The mechanism by which EVs traverse the BBB is still unclear [37]. Banks et al. conducted an intracerebroventricular study to investigate this further with the use of 10 various EV samples from non-cancerous, human, murine, and cancerous cell lines via multiple-time regression analysis. This group found that all 10 varieties of EV crossed the BBB, however, at different rates influenced primarily by lipopolysaccharide (LPS). In 6 of the 10 EV samples, LPS augmented diffusion across the BBB. Further, this group deduced that EVs readily cross the BBB via transcytosis, endocytosis, active efflux, phagocytosis, and carrier-mediated transport [37].

In addition to navigating the limitations of the BBB, choosing the route of administration adds to the challenge of therapeutic success. Some common routes of administration include: (1) systemic administration (such as oral or intranasal); (2) direct administration (such as injection into the cerebrospinal fluid); or (3) drug delivery devices (such as catheters and pumps) [38]. The direct non-invasive route of intranasal administration is commonly used in in vivo studies since this route surpasses the BBB via the olfactory pathway [33]. Gupta et. al. used this route with encapsulated lipid NPs with efavirenz, an antiretroviral, to increase the poor CNS bioavailability [39]. This group found a 150-fold in concentration with intranasal administration of efavirenz in the brain when compared to orally administered efavirenz. Although this route showed promising results, it is restricted by the limited dose amount given through the nasal cavity [33]. Moreover, other animal studies have enlisted the use of other nano delivery systems (i.e., EVs) via intravenous and intraperitoneal routes. Unfortunately, these routes have off-target effects [33,40]. Another way to deliver drugs past the BBB is through direct invasive stereotaxic injections into the CNS [33,41]. This route allows drug concentrations to spread more evenly in the interstitial areas of the brain via convection currents without systemic involvement. As with any invasive procedure, there is a risk of infection in this method.

## 3. Use of Various Artificial/Synthetical Nanoparticles for Targeted Drug Delivery to CNS and Their Limitations

Implementation of synthesized NPs as a noninvasive drug carrier has become a trending field. In recent decades, the development of artificial NPs has served a variety of unmet needs in the drug delivery process. In 1992, INFeD was the first NP therapy approved by the FDA, indicated for iron-deficient anemia. This was followed by Oncaspar, a polymer-protein drug used to treat acute lymphoblastic leukemia and first marketed in 1994. A year later, Doxil was approved as the first liposomal product for the treatment of ovarian cancer, multiple myeloma, and AIDS-related Kaposi’s sarcoma [42]. In the years that followed, different types of NP formulations were approved and now there are thousands of clinical studies on NP therapies registered on the FDA clinical trial database [43]. Current developments are primarily focused on optimizing the physical, chemical, and structural characteristics of polymeric, inorganic, and lipid-based NPs [43].

Synthetic NPs are formed from different materials and have different applications. Polymeric NPs are derived from various polymeric materials, such as poly lactide-co-glycolide, poly(isobutyl cyanoacrylate), poly(ethyl cyanoacrylate), poly(butyl cyanoacrylate), and poly(isohexyl cyanoacrylate) [44]. Inorganic NPs made from metal and silica are applied in drug delivery and medical imaging diagnosis [43,45,46]. Metal NPs have limited solubility, higher toxicity, and therefore, their application is restricted [47]. To date, inorganic NPs have only been approved by the FDA for diseases related to iron deficiency [41]. Lipid-based NPs mainly consists of at least one lipid bilayer surrounding one or more internal aqueous layers. Lipid-based polymeric NPs are mostly taken up and cleared by the mononuclear phagocytic system (MPS). This occurs especially in the liver and spleen where macrophages can engulf NPs and have limited bioavailability and biodistribution [48]. In the CNS, the abundance and wide distribution of microglia is the major component of the MPS. Microglia can play a similar role in the CNS as other MPS cells in various organs clear NPs from circulation [44]. Additionally, receptor-mediated endocytosis by ECs of the BBB also controls the permeability of NPs across the BBB [49,50,51].

To improve BBB permeability, drug loading, circulation modifications to the surface charge, size and components during the synthesis process can be adjusted. However, the modifications can cause some safety issues. Polyethylene glycol (PEG) is a routinely used polymer in NP formulations. Many studies have shown that the addition of PEGylate in a drug enhances therapeutic efficacy and improve drug potency [42]. Young-Sook Kang et al. developed PEGylating immunoliposomes to deliver dopamine to the CNS in a PD rat model. The uptake of dopamine was improved by 8-fold compared to free dopamine [52]. PEGs are common additives in cosmetics, medical treatment, and food [53]. Exposure to PEGylated drugs could introduce the production of an anti-PEG antibody (APA), which is found in around 70% of the general population [54]. APA-mediated drug clearance could also reduce drug concentration in the blood circulation potentially leading to reduced therapeutic efficacy. A clinical study has also shown that APA can circulate in patients for years [55]. It has been observed that instead of the drug itself, the immune reaction triggered by PEG in pegnivacogin, is responsible for severe immediate allergic reactions after its administration. Hence, the measurement of the pre-existing APA level is highly recommended to minimize patients’ risk of a serious allergic reaction [48].

Due to patient heterogeneity and the diversity of biological barriers at different stages of absorption, distribution, metabolism, and excretion, it is a challenging and complicated task to deliver drugs to the CNS in one standardized NP vehicle. The characteristics of NPs vary among each other, and the impact each has on BBB permeability is not well studied. Further, clinical and nonclinical study information regarding targeting drug delivery to the CNS is also very limited. A study on brain metastasis of breast cancer patients under treatment with trastuzumab, glutathione pegylated liposomal doxorubicin hydrochloride, showed that trastuzumab did not effectively hinder the development of brain metastasis in patients receiving trastuzumab compared to the control group [52]. On the other side, in the last two decades, NPs have been a trendy concept and a large number of NP studies for cancer treatment have been published. Among those studies, only 0.7% (median) of the NP-based drug dose was delivered to the targeted tumor sites. This indicates that other than the perspective of the biological environment of the tumor, the characteristics of NPs also limit themselves in encapsulation and in the delivery of drugs to targeted sites [56]. Some other biocompatibility and safety concerns were also raised after the application of NPs, such as hepatotoxicity caused by increased liver enzymes, unclear degradation of NPs in the CNS, and complement system activation by NPs larger than 200 nm leading to an induced inflammatory immune response in the CNS, etc. [57,58].

To develop a drug delivery system with fewer side effects and higher bioavailability, a naturally generated vehicle, EVs, have been brought to the page. EVs are lipid bilayer particles generated by eukaryotes. Initially, it was considered the carrier of metabolites from cells [59]. In the 1990s, it was proven that EVs also play an important role in cell-to-cell communication [60] through carrying signaling molecules; proteins [61,62], DNAs [63,64], and RNAs including mRNAs and non-coding RNA [65]. Due to the potential application of EVs in drug delivery, different technologies have been used to encapsulate drugs in EVs to achieve targeted delivery.

## 4. EV Category & Benefits of EVs in Drug Delivery System and Drug Loading Methods

Depending on the size and biogenesis, EVs have been grouped as exosomes, microvesicles (MVs, also called microparticles), and apoptotic bodies [66]. Although they vary in size and originate from different biogenesis pathways, they carry molecular cargos in a very similar way and mainly consist of lipids, proteins, and nucleic acids [67]. Their capacity to carry molecular cargos may also vary, possibly due to the size and origin of the EVs. EV molecular cargos composition can also be altered by various physiological and pathological conditions and should be exploited before considering EVs for therapeutic purposes [68]. EV sizes range from 30–150 nm—they are also known as exosomes and are generated from the inward budding of endosomes at an early stage and eventually develop into multivesicular bodies (MVBs) [66]. Thereafter, MVBs experience the exocytic fusion with the cell membrane and later on they are released extracellularly. In comparison, microvesicles (MVs) (150–1000 nm in diameter) are directly outward budded from the cell membrane into extracellular space. On the other hand, apoptotic bodies are bigger in size and form through the cell fragmentation and elimination of apoptotic cells. Intriguingly, exosomes and MVs are mainly involved in transmitting molecular information from one cell to another [69]. With the capability of carrying and delivering molecules, EVs have shown their potential for use as a drug delivery system [19]. However, in addition to safety and efficacy, more attention needs to be paid to ensure the maximum drug loading efficiency of EVs and the targeting of these EVs to specific brain organs and cells. Two methods are broadly used for drug loading into EVs: (I) endogenous drug loading and (II) exogenous drug loading.

### 4.1. Endogenous Drug Loading

In this method, the desired cargos including genetic materials, synthetic drugs, and nutraceuticals are simply incubated with cells that tend to release EVs. EV-secreting cells can be chosen depending on the target specific cells or tissues to facilitate the smooth transferring of their cargo via the interaction of similar surface adhesion proteins present on both the EVs and the target cells [70]. Additionally, the uptake of the EVs by the target cells has been achieved by modifying the surface of the EVs using engineering approaches [71]. In the endogenous loading method, upon incubation, the cargo may passively permeate across the cell membrane and can be encapsulated into EVs and released from the cells via a complex and multistage natural mechanism [71]. The lipid bilayer gives EVs an advantage to acquire, store and release drugs to their molecular target. Drug loaded EVs can be taken up by the target cells upon targeted delivery. However, for specific and direct delivery of EVs to the target site, surface modification and delivery route optimization are required. Drug loading into EVs using an endogenous approach is straightforward, however, it requires careful consideration such as drug dosing, cell confluency, the cells passage number, and cell culture conditions.

### 4.2. Exogenous Drug Loading

Another approach of drug loading into EVs is the exogenous drug loading method. This method first involves EV isolation followed by drug loading into the EVs using various mechanical approaches such as incubation, electroporation, sonication, transfection, saponin permeabilization, and mechanical extrusion [72,73]. These methods vary and are dependent on the type and nature of the drugs being encapsulated into the EVs, in addition to the cells being targeted for the EVs mediated drug delivery. Studies showed that exogenous drug loading methods are more efficient at loading the drugs into EVs and significantly enhance the drug loading capacity of EVs [24,74]. It also aids in controlled drug release to the target site upon EV modification [75]. However, there are also disadvantages to loading drugs into EVs exogenously, e.g., EV degradation due to the rigorous procedure involved in the process [19]. Moreover, the stability and bioactivity of EVs could be compromised due to the physicochemical properties of the drugs being loaded. In addition, the lipid bilayer and internal molecular cargo of the EVs could influence the drug loading efficiency. The membrane permeabilizer saponin and hypotonic dialysis could be used to enhance the drug encapsulation efficiency of EVs [74]. Further, studies have shown a comparative analysis of the methods that can yield higher drug loading efficiency [6,42]. In general, the endogenous drug loading approach is highly subjected to the type of drugs and molecules intend to be loaded into the EVs.

## 5. Clinical Trials Evaluating Peripherally Targeted Extracellular Vesicle-Based Therapeutics

EVs offer certain advantages over existing nanomedicine drug delivery platforms: low immunogenicity, less toxicity, better access across the BBB, and inherent homing abilities, all of which make them befitting of drug delivery [65,76]. However, clinical translation of EV therapeutics is still in the early days of development as evidenced by very few early phase clinical trials in recent years [74]. EVs derived from humans and plants are two prominent sources employed in clinical trials, of which plant-derived EV studies are still in their infancy [77]. Human-derived EVs employed in trials are obtained mostly from mesenchymal stem cells (MSCs), dendritic cells, plasma, bone marrow, and patient derived tumor cells [78,79]. Among all the studies, MSC-derived EVs are substantially used as a regenerative medicine in cardiovascular disease, neurological disorders, pulmonary disease, and hepatic illness [80,81,82]. Their widespread application is largely due to their inherent regenerative potential marked by their capacity to promote angiogenesis, induce proliferation, impede inflammatory outcomes, and avert apoptosis [81,83].

Many earlier preclinical studies have shown a prominent role of MSC-derived EVs in the reduction of inflammation, alveolar epithelial apoptosis, and necrosis via modulation of TNF-α, IL-1β, IL-6, IL-10, and iNOS [84,85,86,87,88,89]. Clinical trial NCT04544215 examines mesenchymal progenitor cell (MPC)-derived EVs in critical pulmonary infection caused by Gram-negative bacilli resistant to carbapenems.

The COVID-19 pandemic has pushed doors and opened new avenues in certain research areas [90]. Several trials were registered during the pandemic to explore the untapped potential of EVs in lung pathology associated with SARS-CoV-2 infection [91]. Clinical trials NCT04491240, NCT04276987, NCT04602442, and NCT04313647 are focused on investigating the safety and efficacy of MSC-derived EVs as an aerosol inhalation to harness the cytokine storm in severe hospitalized patients [92]. In NCT04313647, 2 × 10^8^ to 16 × 10^8^ particles of human adipose-derived mesenchymal stromal cell-EVs could be safely administrated into 24 healthy volunteers via a nebulized route without serious adverse events [92]. In yet another study, NCT04493242, intravenous administration of bone marrow-derived EVs was investigated in moderate-to-severe acute respiratory distress syndrome in patients with severe SARS-CoV-2 infection [93]. NCT04493242 is the first clinical study to date using bone marrow MSC-derived exosomes conducted in patients. Exosome formulations were administrated by intravenous injection. The patients achieved significant improvement in neutrophilia and lymphopenia counts and experienced a downregulation of cytokine storm. This study indicated that exosomes could be a promising therapeutic formulation to treat patients with severe SARS-CoV-2 infection [93].

In vitro studies have reported the potential of EVs in the therapy of ophthalmological diseases [94,95,96]. In a dry eye condition associated with chronic Graft Versus Host Disease, Weng et al. reported that MSCs can suppress the inflammation by targeting specific CD8+ CD28− T cells and the same is under examination in clinical trial NCT04213248 [97]. Another clinical trial, NCT03437759, evaluated umbilical MSC-derived EVs for promoting the amelioration of refractory macular holes, a leading cause of central vision loss in the elderly population.

The role of EVs in wound healing has shown that MSC-derived EVs play a significant role in hemostasis by controlling anti-inflammatory processes, proliferation, and remodeling [98,99,100]. In clinical trial NCT02565264, the researchers are studying the effect of plasma-derived EVs on intractable cutaneous ulcers, which are common manifestations in peripheral arterial disease, rheumatic disease, burns, decubitus, and chronic venous insufficiency [101]. Dystrophic epidermolysis bullosa (DEB) is a genetic disorder marked by the mutation in COL7A1 gene and manifested by abnormal blistering of limb skin. Clinical trial NCT04173650 determined the safety and efficacy of MSC-derived EVs application to a DEB wound [102].

The clinical trials NCT03608631 and NCT01294072 employed EVs to deliver their cargo to tumor target sites. In the phase I trial of NCT03608631, they loaded MSC-derived EVs with siRNA against KrasG12D in the treatment of pancreatic cancer with KrasG12D mutation, which metastasizes to other body parts. Numerous studies have reported curcumin as a chemopreventive, antimetastatic, and anti-angiogenic along with its established anti-inflammatory activity [103]. However, its application in clinical therapeutics is largely limited by its poor bioavailability and delivery [104]. A novel approach has been utilized in the trial NCT01294072, in which, plant-derived EVs have been loaded with curcumin, a promising nutraceutical, to improve the bioavailability at target sites in colon cancer.

The following Table 1 summarized the clinical trials we discussed above.

## 6. Drug Delivery to the CNS Cells Using EVs: Promising Drug Delivery Vehicles to the CNS Cells

As NPs are generated from heterogenous material, after administration they face challenges such as clearance, absorption, distribution, metabolism, and excretion. Conversely, we can derive EVs from homogeneous cells to deliver drugs and minimize the unwanted immune response in a non-invasive manner. Lydia et al. loaded siRNA in EVs derived from mice dendritic cells. EVs have been found to be participating in intercellular communication among neurons, astrocytes, ependymoglial, and oligodendrocytes in the CNS [105]. The cross-talk mediated by EVs between those cells can regulate neuronal regeneration and function, which is related to the progression of neurodegenerative diseases such as Alzheimer’s disease (AD) [106], PD [24], HIV-associated neurocognitive disorder (HAND), etc.

The information on clinical trials regarding CNS targeted drug delivery using EVs is very limited. Most of the existing evidence for EV-mediated drug delivery to the CNS comes from pre-clinical studies. The impermeability of the BBB impedes the efficacy of many therapeutic options used to treat or slow the progression of CNS disorders, thus investigation of therapeutic delivery options is pertinent [2,34]. In particular, as seen in the studies given in Table 2, the vehicles of interest for delivery across the BBB are EVs; more specifically cell-derived exosomes. The bioavailability, biocompatibility, increased BBB permeability, and natural source are some of the advantages that exosomes have when compared to other NP delivery options. To successfully deliver across the BBB the studies in Table 2 utilized various EV-loading techniques and routes of administration. Zhuang et. al. incubated EL-4 (murine) exosomes with curcumin or JSI-124 in phosphate buffered saline (PBS); subsequent combinations were separately exposed to sucrose gradient centrifugation, which produced Exo-cur and Exo-JSI124 encapsulated exosomes used for intranasal administration in three different mouse models. Data from this study showed a rapid distribution of 30–100 nm exosomes (primarily in the olfactory bulb brain region) after intranasal administration with the concentration peaking after 1 h and measurable up to 12 h following the initial dose. Exo-JSI124 diminished disease progression and increased tumor apoptosis whereas Exo-cur removed inflammatory CD11b+Gr-1+ cells. Additionally, there were no observable alterations in the murine nasal mucosal epithelial cells, no weight loss, and off-target involvement of the lungs and intestines did occur, but with no apparent toxicity [107]. Haney et al. also used intranasal administration with catalase (9.5 nm) encapsulated in exosomes using 4 different loading techniques [24]. Formulations of exoCAT were created with the following techniques: incubation at room temperature with or without saponin, cycles of freezing and thawing, extrusion, and sonication. The data from this study showed extrusion and sonication resulted equally in the highest loading efficiency. The sonication formulation produced a sustained/prolonged release, and the exosomes significantly increased in size (range 100–200 nm) with extrusion, sonication, and freeze/thaw cycles. Furthermore, the sonication formulation had the highest uptake levels, eliminated ROS in vitro created by macrophages, and indicated greater neuroprotective capability comparison to NP formulations [24]. Qu et. al. studied the effects of dopamine-loaded (via incubation) exosomes versus free dopamine uptake in the brain after intravenous (IV) injection in mice. Data showed that compared to free dopamine, dopamine-loaded exosomes generated fewer toxic effects on neuroblastoma cells, had higher detected levels of dopamine in the brain, and did not cause significant cell apoptosis or the generation of reactive oxygen species (ROS). Moreover, there were no reports of dopamine-loaded exosome related toxicity in the liver, spleen, lungs, or heart; however, mesangiolysis (to the same extent or degree of free dopamine) was suspected due to the breakdown of the exosomes, thus, releasing dopamine into the kidneys [108]. Although studies of EVs show promising therapeutic usage, further investigation is needed to optimize controlled dosage release, EV stability, drug interaction, reduced toxicity at targeted and non-targeted sites, and engineering for clinical feasibility.

## 7. Approaches to Engineer the EVs to Achieve Targeted Delivery to the CNS

EVs have shown their natural targeting feature in vivo and in vitro. The EV was tagged by a peptide, rabies viral glycoprotein (RVG). After systemic administration, EVs targeted the CNS site and succeeded in delivering siRNA efficiently without activation of the inflammatory immune response through the encapsulation of siRNA [29]. Compared to liposomes, EVs have a higher internalization efficiency in target cells [29]. The therapeutic molecule can be loaded in EVs through endogenous loading or exogenous loading. Although the targeting profile of EVs highly depends on their parental cells or the source of EVs. In the process of inward budding of the MVB membrane, some cytosolic components, including RNA, DNA, protein, lipids, and small molecules will be engulfed or encapsulated in EVs or assembled on the membrane of EVs [67]. EV uptake is higher among the same type of cells as their parental cells than of others [112]. Hence, engineering the EVs with a higher affinity to their target cells in the CNS as well as minimizing the uptake of EVs in off-target sites, such as the liver, kidney, spleen, etc. is crucial. Since parent cells determine most EV surface properties, genetic modification during the production process of EVs has been performed; for instance, the fusion protein from cells in EVs with certain molecular-like peptides, antigens, antibodies, etc. [113]. For the targeting profile of EVs, it has been found that EVs have tropism to the cells or tissues where they were derived [114]. Besides genetic modification, direct modification of the parent cells can also be applied. To conjugate fluorescent dyes into EVs, Wang et al. tried metabolic labeling cells via biotinylating without intact EVs [115]. A similar effect is also achieved by fusion of the parent cell membrane with fluorescent-modified liposomes [116]. Modification could also be performed after the isolation of EVs. The fusion of EVs with PEGylated liposomes leads to reduced clearance by macrophages [117]. Inserting a lipid-modified epidermal growth factor receptor (EGFR) binding antibody will provide EV specific targeting to EGFR-expressing cells. Biotinylated PEG-derivatives can also directly attach to the EV surface, which allows EVs to bind Fluorescein isothiocyanate (FITC)-labeled streptavidin [118].

**Figure 2:**
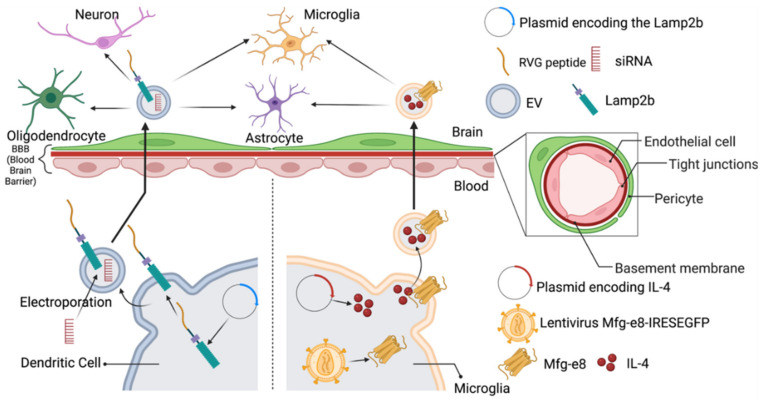
Schematic representation of the engineering of EVs for CNS targeting before isolation. Two studies, as shown in Figure 2, used two different engineering methods before isolation of the EVs from the cell media to deliver therapeutic molecules to the target site in the brain. Dendritic cells were transfected with the targeted gene and fused with RVG peptides to endow the CNS target delivery of EVs, and the therapeutical siRNA was loaded by electroporation before EV isolation. As shown on the right side of the figure, the microglia were infected by a pseudotype virus to overexpress Mfg-e8, and transfected with plasmid coded with IL-4, which has a therapeutical effect on experimental autoimmune encephalomyelitis (EAE) mice. Then, the EVs were produced with a targeting property towards phagocytes in the CNS to treat EAE mice.

Two examples of engineering EVs before EVs isolation are shown in Figure 2. Alvarez-Erviti et al. first demonstrated that the fusion of neuron-specific rabies viral glycoprotein (RVG) peptide to siRNA-carrying dendritic cell-derived EVs expressing Lamp2b led to specific, targeted gene knockdown in in vitro (Neuro 2 A cell lines) and in vivo models [29]. Briefly, dendritic cells were transfected with plasmids encoding the Lamp2b constructs 4 days before exosome production. The EVs released from dendritic cells expressing Lamb2b were fused with the CNS-specific RVG peptide. Later, the glyceraldehyde 3-phosphate dehydrogenase (GAPDH) siRNA was loaded into the RVG EVs by electroporation. The systemic administration of RVG-EVs loaded with GAPDH siRNA resulted in a significant knockdown of GAPDH mRNA in several brain regions but not in other assessed organs. Similarly, to confirm the therapeutic potential of RVG-EVs, the authors loaded BACE1 siRNA, a target for AD, into RVG-EVs. The systemic administration of these EVs to the wild-type mice resulted in a significant BACE1 protein knockdown in the cortical tissue samples. In addition to this, they also observed a significant (55%) decrease in the total β-amyloid 1–42 levels, the main component of the amyloid plaques in pathology of AD.

Microglia account for 10–15% of all cells in the brain, which function as resident macrophages in the CNS [119]. Microglia can engage in diverse cell communication by releasing EVs, which contain small molecules for cell signaling [120]. Casella et al. isolated the EVs from murine microglia after manipulating the expression of Lactadherin (Mfg-e8) and anti-inflammatory cytokine IL-4 in the microglia [121]. After infection with lentivirus Mfg-e8-IRESEGFP (psd44-iGFP-MFG-E8-long, Addgene) in BV-2 cells, Mfg-e8+ BV-2 cells were transfected with a lentiviral plasmid coding for murine IL-4. In vitro, they confirmed that IL-4+ EVs could induce the expression of anti-inflammatory markers, such as arginase-1 (arg1) and chitinase 3-like 3 (ym1) in recipient myeloid cells. Meanwhile, the upregulation of CD206 and arg1 and downregulation of proinflammatory marker iNOS at the mRNA and protein level were observed in the primary microglia. IL-4+Mfg-e8+ EVs could induce more arg1 and ym1 mRNA in recipient cells. In vivo, and intrathecal injection of Mfg-e8+ EVs could diffuse into myeloid cells and astrocytes of the liquor space and spinal cord up to the thoracic region, which indicated that Mfg-e8 increased the uptake of EVs by phagocytes. Moreover, an IL-4+Mfg-e8+ EVs intrathecal injection allowed EVs to directly target meningeal resident cells and could reduce the neuroinflammation in the brain of experimental autoimmune encephalomyelitis (EAE) mice with a sustained release and effect that lasted up to 30 days. By manipulating certain gene expression of parent cells, the delivery of anti-inflammatory cytokines in EVs may prolong the half-life of cytokines and increase their therapeutic effect in the targeted site.

**Figure 3:**
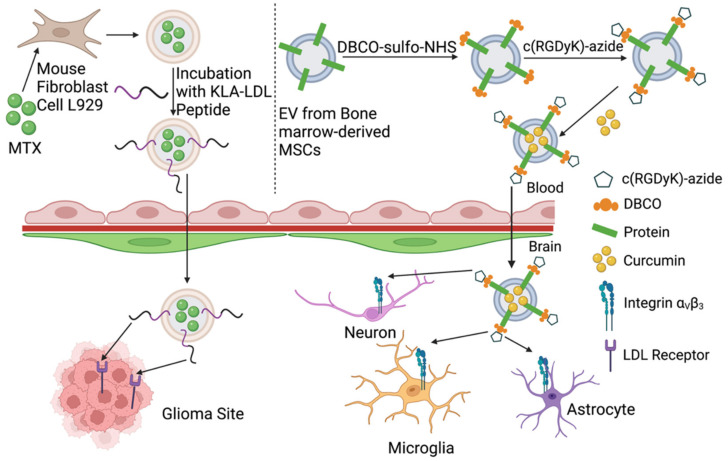
Schematic diagram of engineering of EVs during post isolation to endow EVs with a CNS targeting property. Ye et al. treated parent cells with the drug, Methotrexate (MTX), and fused a peptide onto EVs after isolation. Compared to free drugs, MTX primed EVs delivered more of the active drug across the BBB and reached the glioma site in the mice glioblastoma model. To grant EVs specific targeting capability in the ischemic site in the brain, Tian et al. modified the surface of EVs by click chemistry and loaded EVs with curcumin to treat ischemia, which demonstrated a better safety and efficacy than the control group.

Since phospholipid is rich on the surface of EVs, it provides the possibility to conjugate peptides onto the membrane of EVs during the post isolation of EVs as depicted in Figure 3.

Ye et al. developed a method that methotrexate (MTX, chemotherapeutic drug)-loaded EVs were functionalized with targeted peptides for glioblastoma treatment [122]. Briefly, L929 cells (fibroblast mouse cell line) were treated with MTX for 16 h after irradiation with ultraviolet light to package MTX in EVs derived from L929. After the isolation and purification of EVs, therapeutic [Lys-Leu-Ala (KLA)] and targeted [low-density lipoprotein (LDL)] peptides were added into EVs (MTX@EVs-KLA-LDL) suspension followed by agitation for 3 h at room temperature. They were able to get EVs with around 1.77% conjugation of KLA-LDL and 5.1 ± 0.5% encapsulation efficiency of MTX. The expression of the LDL receptor (LDLR) was higher in glioma BBB and glioma cells compared to normal brain tissues [123,124]. They used a glioma spheroid to verify the penetration and inhibitory effect of MTX@EVs-KLA-LDL in vivo. The glioma spheroids were incubated with MTX@EVs-KLA-LDL at the concentration of 2 μg/mL and 1.6 μM for 12 h and 8 days, respectively. It proved that the conjugation of peptides containing LDLR binding domain could increase the uptake of EVs in LDLR-overexpressed U87 cells and the permeation of EVs in tumor sites. The volume of glioma spheroid after 7-day incubation in control group was 3.97 times that of the initial. But 7-day incubation with MTX, KLA-LDL, MTX@EVs, EVs-KLA-LDL, and MTX@EVs-KLA-LDL were 2.89, 1.95, 1.70, 1.50, and 0.99 times the initial volume, respectively. The MTX@EVs-KLA-LDL group showed a stronger inhibition of the growth of glioma spheroid. Meanwhile, this study first confirmed that EVs-KLA-LDL could cross the BBB more efficiently and distributed more into the glioma related tissues than blank EVs via an IV injection of saline (control), MTX, MTX@EVs, and MTX@EVs-KLA-LDL at days 7, 10, 13, 16 through the tail vein in a glioma-bearing mice model. On day 7, the bioluminescence imaging (BLI) signals were 5.5-fold, 4.7-fold, 1.8-fold in saline, MTX, MTX@EVs groups compared to the control group. This demonstrated that EVs delivered MTX across the BBB with a therapeutic effect. Further, the BLI signal of MTX@EVs-KLA-LDL had a greater antiglioma effect than MTX@EVs. Toxicity and histopathological abnormalities were not observed in all of the groups. Meanwhile, researchers also measured the indicators of liver and kidney function. MTX@EVs or MTX@EVs-KLA-LDL did not affect the kidney function of mice as free MTX. The 48 days’ mid-survival time of mice with MTX@EVs-KLA-LDL was significantly longer than other groups, however, that of the control group, MTX, and MTX@EVs were 28, 30, and 41 days, respectively. Taken together, drug primed EVs with peptides conjugated on the surface can pass through the BBB and have an advanced therapeutic effect on a brain lesion more safely compared to the free drug [122].

Tian et al. conjugated a cyclo(Arg-Gly-Asp-D-Tyr-Lys) peptide [c(RGDyK)] onto the membrane of EVs derived from MSCs by a technology known as biorthogonal copper-free azide alkyne clo-addition (click chemistry) [125]. c(RGDyK) peptide on NPs exhibited tropism to reactivate cerebral vascular ECs in the lesion region of the ischemic brain due to the high affinity of integrin αvβ3 [126,127]. In summary, after EV isolation from MSCs media, there were two steps required to couple the peptide on the EV surface. The first is to use a heterobifunctional crosslinker to incorporate reactive dibenzylcyclootyne (DBCO) groups, and dibenzocyclooctynesulfo-N-hydroxysuccinimidyl ester (DBCO-sulfo-NHS), into the amine-containing molecules on the EV surface. Then, DBCO-conjugated EV (DBCO-Exo) were linked to azide-containing molecules, cyclo(Arg-Gly-Asp-D-Tyr-Lys) peptide(cRGD-EV). In an in vitro study, the results revealed that the uptake of cRGD-Exo in positive αvβ3 HeLa cells was 3-times higher than that of scrambled EV (Scr-EV), while the uptake of cRGD-EV and Scr-EV in the negative αvβ3 HeLa cells did not show a significant difference. An in vivo study of a transient focal cerebral ischemia mice model showed a higher accumulation of cRGD-EV in the ischemic brain, which confirmed the ability of cRGD-EV to target the brain. The finding was due to the strong increase in integrin αvβ3 expression in the ischemia area of the brain, especially on reactive ECs (microglia, neurons, and astrocytes. Then they loaded cRGD-EV with curcumin (cRGD-EV-cur) and injected cRGD-EV-cur into the mice model. After intravenous administration, the inflammation and cellular apoptosis in the ischemic region were suppressed more effectively compared to the curcumin or control EV groups. It indicated that EVs can cross the BBB and enter the brain through IV administration [125]. Based on this study, before drug loading, EVs could be first conjugated with other moieties, which have high tropism to the lesion area of the brain, such as peptides, PEGylated molecules, antibodies, molecules, probes, etc. for targeting delivery in the CNS.

Table 3 compiles several published studies that indicate the modification of EVs for targeted delivery to the CNS.

## 8. Challenges in Targeting EVs Research, Clinical Trials, and Commercial Launch

NPs and nanomedicine delivery to the targeted site played a significant advancement in mitigating critical clinical challenges and the diagnosis of various life-threatening human diseases. Several NP-based treatments have already been approved by the FDA due to their enhanced bioavailability, better pharmacokinetic, and safety profiles. In recent times, naturally occurring EVs have received significant attention in the field of nanotechnology and liposome formulations. However, we still have to resolve a few of the critical challenges that we face today with these particles. Thus, it is worth taking additional scientific efforts to understand the properties behind these vesicles due to their benefits and advantages over the conventional materials in use for liposomal encapsulations. NP formulations were earlier focused upon using lipids, polymers, and cholesterols as an outer scaffold to protect the “payloads” from degradation by serum nuclease and proteases during the parenteral transport to the target site. When the payload cargo carriers have become a key component of site-directed drug delivery systems, EVs were opted for over lipids-polymer hybrid nanoparticles due to their ability to be decorated with numerous surface ligands for recognizing tumor surface receptors. The ability to bear multiple ligands over a single targeting ligand has already been demonstrated as a crucial clinical advantage, as cancer cells can quickly adapt and change their surface receptor expression profiles. In addition, EVs carrying ligand expressions can also be enriched through molecular engineering [29]. Despite the potential promises described above, advances of EV NP delivery systems are still in their infancy due to the key challenges that include, efficient high yielding isolation and purification methods, lacking antigen and drug loading efficiencies, and reproducible transport repeatability of the payload cargo to target cells.

Due to EVs’ advantages over other conventional vesicles, it is worthwhile evaluating those critical challenges in-depth and exploring the potential remediation required to overcome these hurdles. Several modern purification methods were attempted on EVs including immunoaffinity capture, size exclusion chromatography, polymeric precipitation, ultracentrifugation, and microfluidics techniques [128]. Promising remediations are already evident from the brilliant efforts of Lamparski et al. by combining the ultra-centrifugation technique with the ultra-filtration method [129]. Another noticeable EV purification and isolation method on a large scale has caught the attention of clinicians who have long awaited to hear such a promising note. The method they used was very intriguing for large-scale isolation possibilities. The researchers from Qi’s group successfully isolated large quantities of transferrin receptor-expressing exosomes from reticulocytes by incubating with transferrin-coated super magnetic NP and efficiently separated the exosomes via magnetic adhesion [130]. Immediately after, the above technique was combined ingeniously with modern flow cytometry to facilitate the large-scale isolation and purification of other ligands of interest [131]. Other areas of needed improvements for EVs are also currently underway in research laboratories and preliminary positive outcomes are eminent.

**Figure 4:**
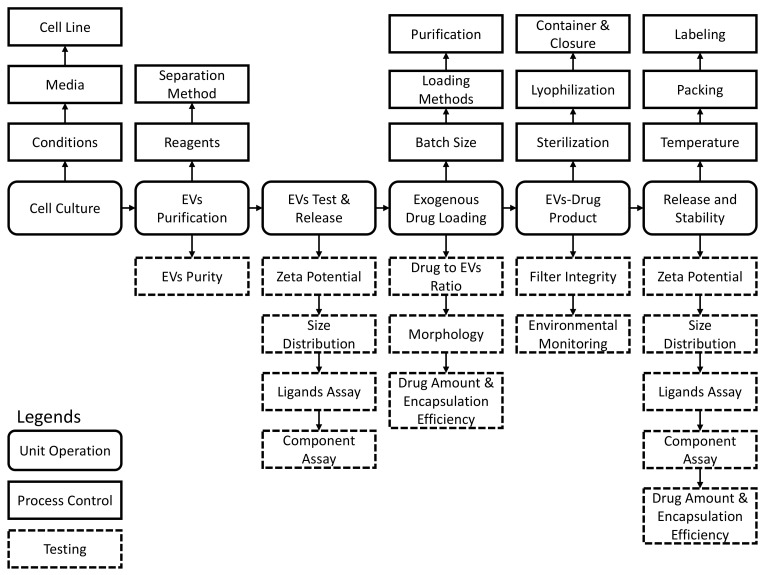
A conceptual process flow chart describing the unit operations for EV drug products. This flow chart summarizes the key steps to maintain the consistency of safety, efficacy, and quality in EV drug products based on the regulatory requirements of the FDA and the feasibility in manufacture process.

It is also critical that the abovementioned scalable isolation and purification methods, once established, should be conducted in accordance with the federal code of regulations set forth by the FDA to ensure batch-to-batch consistency of purity, impurity profile between batches, and usage of materials and components that are qualified and approved as the end product is for human patient use. The nature of EVs is the very first obstacle since cells are involved. If cells from plants or animals are used to absorb drug substances, then secrete EVs with encapsulated drugs, they should be considered as biologics [132]. Even though EVs only serve as a carrier, the nature of the processes disqualifies them from small molecule drug products. Conversely, if EVs are extracted and purified as excipients, then drug substances are loaded into EVs through external agitation, these products may be treated in a similar way as liposome drug products [133]. The first method is called endogenous EV drug loading, while the second approach is named exogenous EV drug loading [134]. Based on current regulatory policies, EV drug products will face various difficulties in manufacturing, quality control, and quality assurance as shown in Table 4 and Figure 4.

According to the current FDA’s guidance for the industry regarding liposome drug products, the FDA requires components of whole drug products, including liposome components, which should also apply for EV drug products considering their similar structure and components [133]. Very few research articles have investigated the components of EVs, and they only disclose the saucing cells. Further, EVs may have a wide spectrum of morphology and lamellarity [135]. Size distribution is another concern as EVs may vary from 50 nm to 1 µm in size [134]. Since EVs’ biggest advantage is their targeting capability, the targeting ligands need to be intact throughout the manufacturing process. Other parameters that need to be monitored include particle size, EV membrane integrity (morphology), drug encapsulation, etc. All these questions and concerns must be addressed through a series of in-process testing to ensure the functions of EVs remain intact. Further, EV drug products can only be delivered via injection. Hence, the final products must comply with requirements for sterile parenteral products. The manufacturing process needs to be either validated or not validated but with strict testing to ensure the quality of products.

## 9. Conclusions and Future Perspectives

EVs are emerging as a promising drug delivery vehicle over artificial NP drug delivery due to their intrinsic properties of biocompatibility, low immunogenicity, and stability in circulation. For any drug delivery strategy to be successful, it should have the desired effect at the target site and minimal off-target effects. Due to the presence of specific surface proteins or the type of proteins they carry based on the cells of the origin, EVs are being explored to achieve targeted drug delivery, particularly across epithelial barriers such as mucosa or the BBB. A major obstacle in treating most CNS disorders is the impermeability of drugs across the BBB. Due to the ability of EVs to cross the BBB, EVs are being utilized as efficient drug delivery vehicles to treat various CNS disorders. Various methods, including the surface modification of EVs, conjugation of peptides to EVs, etc. have been employed to achieve the targeted delivery of EVs and minimize the off-target effects. To date, most clinical trials on EVs are undergoing with very limited published information on their current progress or results. Translating these modified EVs into clinics, as discussed above, has major challenges, and more research is needed as they have a long way to go to achieve their full therapeutic potential.

The synthetic DDSs, such as NPs [136,137,138], liposomes [139,140,141,142], dendrimers [143,144,145], micelles [146,147,148], nanocapsules [149,150,151], nanosponges [152,153], peptide-based nanoparticles [154], etc., have good drug encapsulation efficiency (EE), drug loading (DL) capacity, flexibility in functionalization, easy/robust production, and theragnostic functions. However, conjugation of a PEG polymer, peptide, antibody, etc. may develop drug tolerance [54,127] and even cause allergy [48]. On the other hand, EV DDSs have the advantage of improved cellular uptake, intrinsic targeting capacity, and low immunogenicity biocompatibility. EVs have shown an innate ability to target tumor and immuno-evasive properties. Various types of EV-hybrid NPs have been engineered for targeted cancer therapy. EVs derived from macrophages, microglia, and MSCs have been shown to specifically accumulate in the inflamed brain and can deliver therapeutic proteins to the brain [23,126,130]. To improve the consistent EE, DL, quality, safety, and efficacy profile of EV drug products, one approach to achieving EVs’ full targeted therapeutic potential would be combining EVs from aforementioned sources with conventional DDSs, NPs, or liposomes to load drugs and treat various CNS disorders. Therefore, an EV-hybrid system derived from the combination of conventional DDSs and EV components is expected to pose both beneficial properties.

## Figures and Tables

**Table 1:** Clinical trials evaluating peripherally targeted EV-based therapeutic.

Sr No	Clinical Trial	Status	Phase	Subject	Indication	EV Source	Target Sites	EV Manipulation	Reference
1.	Evaluation of Safety and Efficiency of Method of Exosome Inhalation in SARS-CoV-2 Associated Pneumonia. (COVID-19EXO)	Completed	1/2	30	COVID-19	MSC ^1^-derived	Lungs	NA	NCT04491240
2.	A Pilot Clinical Study on Inhalation of Mesenchymal Stem Cells Exosomes Treating Severe Novel Coronavirus Pneumonia	Completed	1	24	COVID-19	Allogenic adipose MSC ^1^-derived	Lungs	NA	NCT04276987
3.	Safety and Efficiency of Method of Exosome Inhalation in COVID-19 Associated Pneumonia (COVID-19EXO2)	Enrolling by invitation	2	90	COVID-19	MSC ^1^-derived	Lungs	NA	NCT04602442
4.	COVID-19 Specific T Cell Derived Exosomes (CSTC-Exo)	Active, not recruiting	1	60	COVID-19	COVID-19 specific T-cells derived	Lungs	NA	NCT04389385
5.	Extracellular Vesicle Infusion Treatment for COVID-19 Associated ARDS (EXIT-COVID19)	Completed	2	120	COVID-19 Associated ARDS	Bone marrow derived	Lungs	NA	NCT04493242
6.	A Clinical Study of Mesenchymal Progenitor Cell Exosomes Nebulizer for The Treatment of Pulmonary Infection	Recruiting	1/2	60	Drug resistant pulmonary infection	MPC ^2^-derived	Lungs	NA	NCT04544215
7.	A Tolerance Clinical Study on Aerosol Inhalation of Mesenchymal Stem Cells Exosomes in Healthy Volunteers	Completed	1	24	Safety and tolerance	Allogenic adipose MSC ^1^-derived	Lungs	NA	NCT04313647
8.	A Clinical Study of Mesenchymal Stem Cell Exosomes Nebulizer for the Treatment of ARDS	Not yet recruiting	1/2	169	Acute Respiratory Distress Syndrome	Allogeneic human MSC ^1^-derived	Lungs	NA	NCT04602104
9.	Effect of UMSCs Derived Exosomes on Dry Eye in Patients With cGVHD	Recruiting	1/2	27	Dry Eye	Umbilical MSC ^1^-derived	Eyes	NA	NCT04213248
10.	MSC ^1^-Exos Promote Healing of MHs	Active, not recruiting	Early Phase 1	44	Macular Holes	MSC ^1^-derived	Retina-Eyes	NA	NCT03437759
11.	Evaluation of Adipose Derived Stem Cells Exo. in Treatment of Periodontitis (exosomes)	Recruiting	Early Phase 1	10	Periodontitis	Adipose- stem-cell-derived	Gums-oral cavity	NA	NCT04270006
12.	Edible Plant Exosome Ability to Prevent Oral Mucositis Associated with Chemoradiation Treatment of Head and Neck Cancer	Active, not recruiting	1	60	Oral Mucositis in Head and Neck Cancer	Grape derived	Oral cavity	NA	NCT01668849
13.	MSC ^1^ EVs in Dystrophic Epidermolysis Bullosa	Not yet recruiting	1–2	10	Dystrophic Epidermolysis Bullosa	Allogeneic MSC ^1^-derived	Integument	NA	NCT04173650
14.	Effect of Plasma Derived Exosomes on Cutaneous Wound Healing	Unknown	Early Phase 1	5	Intractable cutaneous ulcers	Plasma derived	Integument	NA	NCT02565264
15.	Use of Autologous Plasma Rich in Platelets and Extracellular Vesicles in the Surgical Treatment of Chronic Middle Ear Infections	Recruiting	2–3	100	Otitis Media	Plasma derived	Middle ear	NA	NCT04761562
16.	Effect Of Microvesicles and Exosomes Therapy on Β-Cell Mass in Type I Diabetes Mellitus (T1DM)	Unknown	2/3	20	Diabetes Mellitus Type 1	Umbilical cord-blood derived MSC ^1^-derived	Pancreas	NA	NCT02138331
17.	iExosomes in Treating Participants with Metastatic Pancreas Cancer with KrasG12D Mutation	Recruiting	1	28	Metastatic Pancreatic Adenocarcinoma, Pancreatic Ductal Adenocarcinoma	MSC ^1^-derived	Metastatic Pancreatic cancer cells	loaded with siRNA against KrasG12D	NCT03608631
18.	Study Investigating the Ability of Plant Exosomes to Deliver Curcumin to Normal and Colon Cancer Tissue	Recruiting	1	35	Colon Cancer	Plant derived	Colon	Loaded with curcumin	NCT01294072

^1^ MSC: Mesenchymal stem cell; ^2^ MPC: Mesenchymal Progenitor Cell.

**Table 2:** EVs drug delivery systems in CNS cells.

EVs Source	Encapsulated Agents	Target	Goal	Outcome
EL-4 T cells [107]	1. Curcumin 2. JSI-124 (cucurbitacin I)	Microglial cells	To show that the intranasal administration of curcumin and JSI-124 encapsulated in exosomes can pass the BBB and prevent microglial cell activation induced by lipopolysaccharide, delay experimental autoimmune encephalomyelitis disease, and inhibit tumor progression in vivo.	Intranasal administration of curcumin and JSI-124 encapsulated in exosomes showed a rapid uptake by microglial cells and provided neuroprotection. This approach has the potential to be a non-invasive treatment option in brain inflammatory-related diseases
Mouse macrophage cell line (Raw 264.7) [24]	Catalase (antioxidant)	Neurovascular unit: 1. Endothelial cells 2. Neurons 3. Astrocytes	To show that intranasal administration of exosomes loaded with catalase may protect catalase enzymatic activity, decrease immunogenicity, and extend blood flow time in a Parkinson’s Disease mouse model.	Intranasal administration of exosomes loaded with catalase showed significant neuroprotective effects (in vitro and in vivo) and thus potentially an applicable treatment strategy for inflammatory and neurodegenerative disorders.
Blood of Kunming mice [108]	Dopamine	Brain epithelial cells	Show how loading blood derived exosomes with dopamine may increase distribution past the BBB and thus a more effective drug delivery approach compared to conventional treatment options.	Blood derived exosomes were delivered across the BBB via the transferrin-TfR interaction, thus dopamine distribution increased >15-fold and toxicity significantly decreased compared to free dopamine.
Bone marrow-derived mesenchymal stem cell modified with rabies virus glycoprotein (RVG) [109]	microRNA-124	The ischemic cortex of brain	Investigate if loading miR-124 into RVG-modified exosomes can safeguard against cortical ischemia.	miR124 shown to be neuroprotective and to lead neuron remodeling via promotion of neurogenesis, thus can be considered a promising gene therapy approach for ischemic injury.
Brain endothelial bEND.3 cells [110]	Vascular endothelial growth factor small interfering RNA (VEGF siRNA)	Neuronal glioblastoma-astrocytoma U-87 malignant glioma cells	Given siRNA’s therapeutic potential, to test if brain endothelial cell-derived exosomes can cross the BBB in zebrafish with U-87 malignant gliomas (MG) glioblastoma to deliver siRNA.	In glioblastoma-astrocytoma U-87 MG cells expression of vascular endothelial growth factor (VEGF) RNA and protein levels were inhibited by the exosomal delivery of siRNA.
1. Brain neuronal glioblastoma-astrocytoma U-87 MG cells 2. Brain endothelial bEND.3 cells 3. Neuroectodermal tumor PFSK-1 cells 4. Glioblastoma A-172 cells [111]	1. Rhodamine 123 2. Paclitaxel 3. Doxorubicin	Neuronal glioblastoma-astrocytoma U-87 malignant glioma cells	Evaluate drug delivery across the BBB based on particle size, morphology, total protein, and transmembrane protein markers.	Brain endothelial bEND.3 exosome drug delivery performed best compared to the others. bEND.3 exosome success was attributed to the high expression levels of CD63.

**Table 3:** Engineering of EVs for targeted delivery to the CNS.

**Engineering Method**	**Target Site**	**Outcomes**	**Potential Application on CNS Targeting**	**Reference**
**Engineering EV Parent Cells before the Isolation of EVs**				
Lamb2b plasmid was transfected into dendritic cells 4 days before EVs isolation. RVG peptides were cloned into extra-exosomal N terminus of Lamp2b. After EV isolation, load EVs with BACE1 siRNA via electroporation	Acetylcholine receptor in brain	Intravenous (IV) injection of RVG-targeted EVs loaded with BACE1 siRNA can knockdown mRNA (60%) and protein (62%) expression of BACE1 in the brain. Uptake was not observed in other off-target organs in mice.	Delivery of gene therapy in CNS for neurodegenerative diseases	[29]
EVs were generated by BV-2 microglia cells infected with the lentivirus Mfg-e8-IRES-EGFP to overexpress Mfg-e8 and transfected with a lentiviral plasmid coding for IL-4.	Phagocytes in brain	After cisterna magna injection of IL-4+Mfg-e8+ EVs into mice, EVs could target phagocytes and anti-inflammatory markers. Chitinase 3-like 3 (ym1) and arginase-1 (arg1) were upregulated in the CNS, which decrease neuroinflammation and brain damage.	Engineer anti-inflammatory molecules to treat neuroinflammatory diseases	[121]
**Engineering EV after EV isolation**				
Conjugate cyclo(Arg-Gly-Asp-D-Tyr-Lys) peptide [c(RGDyK)] onto EVs derived from mesenchymal stromal cell (MSC) surface using click chemistry. Curcumin(cur) was incorporated in the cRGD-Exo for 5 min at RT	Target site: cerebral vascular endothelial cells in the brain	IV administration of cRGD-EXO-cur could successfully suppress inflammation and cellular apoptosis in the ischemic brain in mice	Load therapeutic agents into cRGD-Exo to target the lesion region of the brain	[125]
EVs derived from L929 cells were loaded with methotrexate and conjugated with [Lys-Leu-Ala (KLA)], containing an ApoA-I mimetic sequence, and [low-density lipoprotein (LDL)], phospholipids, by agitation at room temperature for 3 h.	Glioma spheroid in the brain	EVs-KLA-LDL were injected intravenously. They crossed the BBB more efficiently than the control EV and an inhibition of glioma spheroid growth after administration of EVs-KLA-LDL was observed, resulting in improved survival in mice models.	Conjugation of peptides onto EVs surface during post-isolation modification can improve penetration across the BBB of EVs and their target binding for brain tumor tissue, which improves the therapeutic effect of drugs.	[122]

**Table 4:** Release tests for EV drug products (USP).

Purpose	USP Chapter	Test
Release	N/A	EVs and ligands assay
	N/A	Drug substance assay
	<71>	Sterility
	<785>	Osmolality
	<467>	Residual organic solvents *
	<281>	Residue on ignition
	<731, 921>	Loss on drying for lyophilized products
	<790>	Visible particulate inspection
	<61>	Microbial enumeration
	<791>	pH
	<85>	Bacterial endotoxins
	<788>	Particulate matter for injection
	<1207>	Uniformity of dosages

* Only needed if organic solvents are used during the process.

## Data Availability

No new data were created or analyzed in this study. Data sharing is not applicable to this article.
